# Stress Processes, Acculturation, Gender and Memory Performance: A HABS-HD Study

**DOI:** 10.1093/geroni/igaf122.2467

**Published:** 2025-12-31

**Authors:** Jordana Breton, Elizabeth Muñoz

**Affiliations:** The University of Texas at Austin, Austin, Texas, United States; University of Texas at Austin, Austin, Texas, United States

## Abstract

Latinos are at greater risk for dementia compared to non-Latino White adults, with women being at greater risk compared to men. Stress is a modifiable risk factor linked to cognition across gender; however, men and women experience stress differently. Higher acculturation is linked to better cognition, but it is unclear if it mitigates the negative effects of stress on cognition or whether these associations differ by gender, as acculturation levels differ by gender. We examined the interactive effects between stress exposure/appraisal (SE/SA) and acculturation on learning and memory in 260 men and 525 women who participated in the Health and Aging Brain Study: Health Disparities (Mean age= 63.16; range = 50-91). Participants self-reported gender, SE and SA, and acculturation. Outcomes included total learning and delayed recall trials from the Spanish English Verbal Learning Test. Gender stratified linear regression models examined SE/SA x acculturation on learning/memory outcomes. Gender stratified linear regression models revealed that higher SA was linked with better learning only for women with high acculturation (B = -0.265, SE = 0.11, p = 0.016). Higher SA was associated with better memory for men with high acculturation, but lower memory for men with low acculturation (B = -0.432, SE = 0.171, p = 0.012). There were no significant SE x acculturation interactions on learning/memory across gender. Findings suggest that high acculturation may be protective against the negative effects of SA for learning in women and memory in men, elucidating the heterogeneity in the protective role of sociocultural factors among Latinos.

